# P-640. Not All Viruses Are Equal: A Comparative Study Of COVID-19, Influenza A, RSV And Work Absenteeism

**DOI:** 10.1093/ofid/ofaf695.853

**Published:** 2026-01-11

**Authors:** Joy Abou Farah, Alexia El Khoury, Chris D Ladikos, Zainab Albar, Jay Krishnan, Elie Saade

**Affiliations:** Case Western Reserve university, Cleveland, OH; Case Western Reserve university, Cleveland, OH; University hospitals, Cleveland, Ohio; Case western reserve university school of medicine, cleveland, Ohio; University hospitals, ID clinical trial unit, Cleveland, Ohio; Case Western Reserve University, Cleveland, OH

## Abstract

**Background:**

Respiratory viral illnesses significantly impact workforce productivity and absenteeism, yet few studies have explicitly compared the relative impacts of COVID-19, influenza A, and RSV within occupational settings. This study compares virus-specific impacts on work absenteeism among working-age adults to guide preventive strategies and workplace policies.

**Figure:**
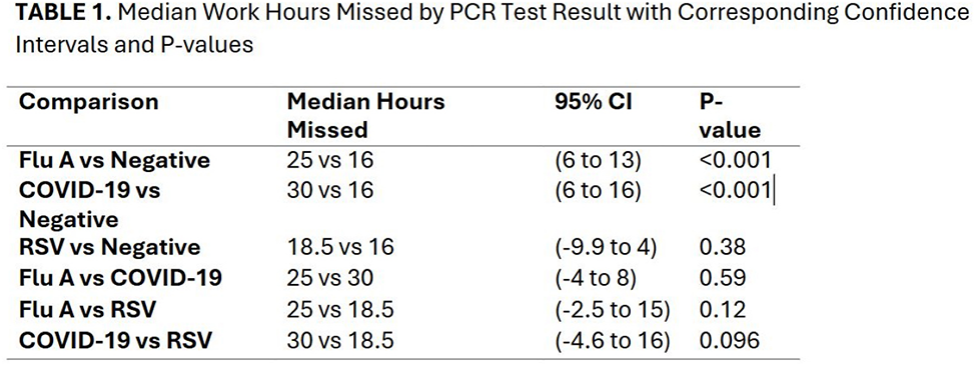

**Methods:**

We conducted a prospective observational study on 404 adults aged 18–65 y in emergency rooms and urgent cares for influenza-like illness (ILI) during the 2024–2025 respiratory season. Participants initially completed a baseline survey that included self-reported vaccination status and underwent PCR testing for COVID-19, influenza A, and RSV. Subsequently, they completed a follow-up survey two weeks later to report on work hours missed and the impact of illness until recovery. Virus-specific impacts were analyzed using Wilcoxon rank-sum tests with Bonferroni correction to account for multiple comparisons. Multivariable generalized linear models (GLMs) with a Gamma distribution and log link function were constructed to evaluate independent associations between virus type and missed work hours, adjusting for age, sex, education level, remote work capability, and virus type (PCR result).

**Results:**

COVID-19-positive participants missed more work hours (median: 30 hours) than those who tested negative (16 hours; p< 0.001). Influenza A cases showed significant absenteeism (27.6 hours; p< 0.001), while RSV-positive cases showed no significant difference (19.9 hours; p=0.38). Adjusted analyses confirmed increased absenteeism for (11.95 additional hours; p< 0.001) and influenza A (8.6 additional hours; p< 0.001). Remote work capability significantly reduced missed work hours (IRR 0.57 for Flu A, IRR 0.61 for COVID-19; p< 0.001).Remote work capability significantly reduced missed work hours (IRR 0.57 for Flu A, IRR 0.61 for COVID-19; both p< 0.001).

**Conclusion:**

COVID-19 and Influenza A increase workforce absenteeism compared to test-negative ILI cases. Flexible workplace policies, particularly remote work capabilities, are critical to reducing the occupational impact of respiratory viral illnesses.

**Disclosures:**

All Authors: No reported disclosures

